# Optical Fiber-Integrated Metasurfaces: An Emerging Platform for Multiple Optical Applications

**DOI:** 10.3390/nano12050793

**Published:** 2022-02-26

**Authors:** Qiancheng Zhao, Weihao Yuan, Jiaqi Qu, Zhi Cheng, Gang-Ding Peng, Changyuan Yu

**Affiliations:** 1 Photonics Research Center, Department of Electronic and Information Engineering, The Hong Kong Polytechnic University, Hong Kong, China; qiancheng.zhao@polyu.edu.hk (Q.Z.);weihao.yuan@connect.polyu.hk (W.Y.); jiaqi.qu@connect.polyu.hk (J.Q.); chengzhi@tju.edu.cn (Z.C.); 2 Photonics & Optical Communication, School of Electrical Engineering, University of New South Wales, Sydney, NSW 2052, Australia; g.peng@unsw.edu.au

**Keywords:** metasurface technology, nanostructures, lab-on-fiber, optical fiber metasurfaces

## Abstract

The advent of metasurface technology has revolutionized the field of optics and photonics in recent years due to its capability of engineering optical wavefronts with well-patterned nanostructures at subwavelength scale. Meanwhile, inspired and benefited from the tremendous success of the “lab-on-fiber” concept, the integration of metasurface with optical fibers has drawn particular interest in the last decade, which establishes a novel technological platform towards the development of “all-in-fiber” metasurface-based devices. Thereby, this review aims to present and summarize the optical fiber-integrated metasurfaces with the current state of the art. The application scenarios of the optical fiber metasurface-based devices are well classified and discussed accordingly, with a brief explanation of physical fundamentals and design methods. The key fabrication methods corresponding to various optical fiber metasurfaces are summarized and compared. Furthermore, the challenges and potential future research directions of optical fiber metasurfaces are addressed to further leverage the flexibility and versatility of meta-fiber-based devices. It is believed that the optical fiber metasurfaces, as a novel all-around technological platform, will be exploited for a large range of applications in telecommunication, sensing, imaging, and biomedicine.

## 1. Introduction

Optical fiber has long been a well-established medium since the first demonstration of silica-based fiber with low-loss transmission less than 20 dB/km in the 1970s [1]. Benefiting from its extraordinary features such as perfect light guiding, light volume, chemical inertness, and immunity to electromagnetic interference, a myriad of optical fiber-based applications have been realized, which has greatly revolutionized the optical sensing [2,3,4,5,6] and telecommunication industry [7,8,9] in the last five decades. Despite the tremendous success of optical fiber technology, it turns out that there remain several challenges that obstruct the further progress of optical fiber-based devices. The optical properties such as the propagation direction of guided modes, amplitude, mode profile, polarization states, are hardly to be altered after the fiber drawing fabrication process. Moreover, the divergence of output transmitted light and chromatic dispersion of the optical fiber also limits the practical applications in long-haul transmission systems. In this regard, the recent concept of “lab-on-fiber” has opened up a new pathway to functionalize conventional fibers for multiple applications (e.g., environmental sensing, biomedicine, clinical diagnosis [10,11,12]), with the enhanced light-matter interactions introduced by the dielectric or metallic nanostructured patterns embedded on the facet of optical fibers. Undoubtedly, the lab-on-fiber paradigm has greatly boosted the creation of novel plug-and-play “all-in-fiber” devices that are accessible in various application scenarios. However, most of the conventional nanostructures are formed by artificial 3D metamaterials (e.g., “meta-atoms”). The fabrication process of 3D metamaterials is labor-intensive and costly, which brings complexity and impediments for practical optical applications.

Fortunately, the appearance of metasurface technology in the last decade has further brought disruptive innovations to the nanophotonic field. The metasurfaces, based on the phase discontinuities with 2D counterparts of metamaterials, could flexibly engineer the properties (e.g., phase, amplitude, and polarization) of the incident light. Therefore, a number of applications have been surged, including beam steering [13], aberration-free focusing metalenses [14], polarization control [15,16], holography [17,18,19], and imaging [20,21,22]. Inspired by the lab-on-fiber technology, the integration of metasurfaces on optical fibers, as a novel landmark in the lab-on-fiber realm, has attracted enormous attention in recent years. The flexibility, biocompatibility, and mechanical robustness have made optical fibers excellent platforms to be linked to metasurface technology, which is expected to leverage the functionalities pertaining to optical fiber technologies to be applied to real-world scenarios.

In this article, the basic physics and working principles of metasurfaces are explicitly elucidated in the first part, which are supported as the theoretical basis for the following optical fiber-integrated metasurfaces. Subsequently, the latest applications of optical fiber metasurface-based devices, depending on their specific design methods and application scenarios, have been classified accordingly and discussed comprehensively. Moreover, the corresponding fabrication techniques of optical fiber metasurfaces have been presented with the comparison of the merits of each technique. Furthermore, the potential challenges and future prospects in the field of optical fiber metasurfaces are also outlined, which may shed some light on the efficient bridging between fiber-optic technology and the “flat” photonics with a plethora of potential applications with high compactness, compatibility, and efficiency.

## 2. Basic Concepts and Principle of Metasurfaces 

**Figure 1:**
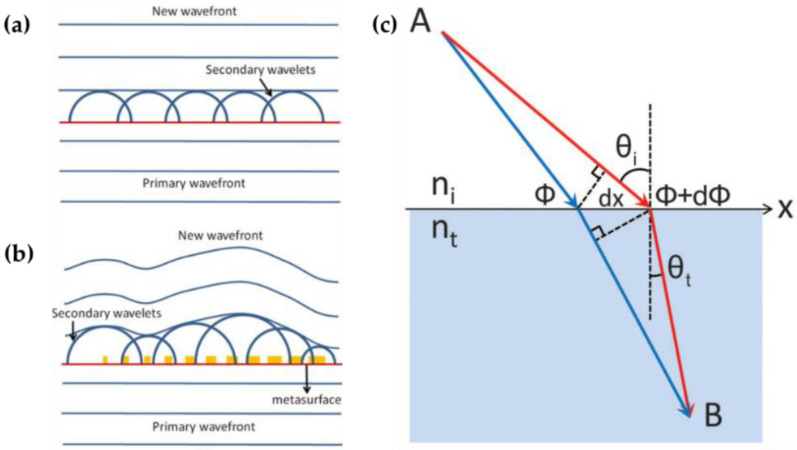
Schematics showing the Huygens’s principle with optical wavefront impinging on (**a**) nonstructured surface, and (**b**) a metasurface. Reprinted with permission from Ref. [23]. Copyright 2015 IEEE Photonics Society. (**c**) Schematic of derived generalized Snell’s law of refraction. Reprinted with permission from Ref. [13]. Copyright 2011 American Association for the Advancement of Science.

Metasurfaces are typically made up of arrays of antennas that are spatially at subwavelength scale with varying geometric parameters. The light propagating through metasurfaces will undergo varying spatially optical responses and thus be shaped by the phase discontinuities (defined as the abrupt phase change over a distance compared to wavelength). The working principle of metasurfaces was systematically demonstrated by Yu and Capasso in 2011, where the generalized Snell’s laws were formulated with the introduction of the concept of phase discontinuities [13]. Briefly, the working principle of metasurface can be explained from the perspective of Huygen’s principle: Each point on the interface can be regarded as an independent source and generated as a sphere wavelet, and a new wavefront is thus created by the interference of these wavelets. For a regular nonstructured surface, there is no change of propagation direction for the incident light. However, in the case of inhomogeneous metasurface consisting of arrays of resonators (e.g., antenna, nanopillars, nanobricks, etc.), the wavefront will be reconstructed due to the distinct phase response of these spatially arranged resonators, as indicated by Figure 1a,b. To further analyze the phenomenon of reflection and refraction of light interacting with metasurfaces, Fermat’s principle can be applied, stating that two infinitesimally close paths should have optical phase difference of zero (so-called “stationary phase”). These optical paths include the inherent propagation phase and the phase change induced at the interface, as shown in Figure 1c.

Consequently, by applying the stationary phase condition on the phase gradient metasurface, Snell’s law can be extended and generalized with an additional item related to phase gradient, as indicated in Equation (1)

(1) $$sin\left(\theta_{t}\right)*n_{t}-sin\left(\theta_{i}\right)*n_{i}=\frac{\lambda }{2\pi }\frac{d\varphi }{dx}$$

where *θ_t_* and *θ_i_* are the refraction and incident angles, respectively, and *n_t_* and *n_i_* are the refractive indices of the two media. The constant phase gradient, denoted as *dφ/dx*, is determined by the specific geometries and spatial arrangements of the resonators. Equation (1) also implies that the refracted beam can be directed arbitrarily. Furthermore, under the condition *n_t_* < *n_i_*, the critical angles under the total internal reflection condition can be satisfied and derived, as:

(2) $$\theta_{c}=\arcsin\left(\pm \frac{n_{t}}{n_{i}}-\frac{\lambda }{2\pi n_{i}}\frac{d\varphi }{dx}\right)$$

Similarly, in the case of reflection with gradient metasurface, the generalized Snell law can be rewritten as:

(3) $$\sin\left(\theta_{r}\right)-\sin\left(\theta_{i}\right)=\frac{\lambda }{2\pi n_{i}}\frac{d\varphi }{dx}$$

where *θ_r_* is the reflection angle. It is seen from Equation (3) that the anomalous reflection is no longer equal to the incident angle, which differs significantly from the conventional specular reflection. In addition, Equation (3) indicates that there exists a critical angle beyond which the reflected beam becomes evanescent, which is expressed as:

(4) $${\theta^{\prime }}_{r}=\arcsin\left(1-\frac{\lambda }{2\pi n_{i}}\left|\frac{d\varphi }{dx}\right|\right)$$

As seen from Equations (1)–(4), the light manipulation is closely associated with the phase gradient induced by the optical resonators constituting the 2D metasurfaces. The optical resonators can be selected from a wide range such as dielectric resonators, quantum dots, nano-crystals, and plasmonic antennas. However, it should be noted that the resonators have to satisfy the following requirements: (1) they should have subwavelength geometric parameters to be arranged at subwavelength scale with limited transmission loss. (2) The phase modulation of these resonators should cover the entire 2π range. (3) The scattered optical amplitude should be uniform and large across the metasurface array. Based on the principles and physics of metasurfaces, Yu and Capasso et al. have successfully pioneered a variety of flat optical components based on metasurfaces, including metalens [24], quarter-wave plates [15], vortex plates [25], and holograms for vortex beam generation [26]. In particular, successful implementation of the metasurface-based collimating lens on the facets of semiconductor lasers to control the far-field laser emissions (e.g., divergence angle [27], output power [28]) has shown promising inroads towards the production of metasurface-integrated devices. This would be of great value to propel the optical fiber-integrated metasurfaces, because in both platforms the light propagation is well confined in the optical waveguide.

## 3. Applications of Optical Fiber Meta-devices 

Illuminated by the successful integration of metasurface with the well-established platform of semiconductor lasers for wavefront engineering, there is sufficient grounds that the metasurface technology could also bring new features to conventional optical fibers to launch a novel class of all-fiber devices and components: (1) the metasurface arrays can be readily patterned on the facets (e.g., end face of the fiber core, the side face of D-shaped fiber) of optical fibers to interact with either the confined or evanescent fields [29,30,31]. (2) The compact resonators of metasurface nanostructures on the optical fiber platforms can have strong interactions with either the electric field or magnetic field of the guided light, thus controlling the optical impedance with modified transmission or reflection properties. (3) The integrated metasurface with high refractive index materials is capable of modulating the optical properties of the guided mode, including the phase, amplitude, and wavevector. Therefore, optical fiber metasurface-based devices have sprung up during the last decade and have been exploited in many strategic applications, ranging from optical processing and communication to environmental sensing, biomedicine, and security. In the following, the specific application scenarios, design methods, and brief physics of optical fiber-integrated metasurface-based devices are categorized, reviewed, and discussed accordingly.

### 3.1. Function of Light Beam Focusing 

One of the most pronounced and repetitively reported functions of fiber-integrated metasurface is the light beam, focusing on fiber guided fundamental mode. Typically, to transfer the input plane wavefronts to the focused spherical ones, the phase retardation of the predesigned metalens should follow the hyperbolic phase profile, which is expressed as [32,33]:

(5) $$\varphi (x,\;y)=-\frac{2\pi }{\lambda }(\sqrt{f^{2}{+x}^{2}{+y}^{2}}-f)$$

where (*x*, *y*) refers to the spatial coordinate in which each unit cell of the metalens is located, *f* is the designed focal length, and *λ* is the operating wavelength. To realize the target hyperbolic phase distribution, several phase modulation methods for the spatially distributed nanopillars can be considered. Depending on the polarization sensitivity of the incident beam, the phase modulation methods can be classified into two types: one is the propagation phase modulation [34], in which the phase difference is mapped by square cylinders or cylinders utilizing varying side lengths or diameters. Each nanofin can be regarded as a waveguide and thus introduce the waveguiding effect as the following [35]:

(6) $$\varphi_{\mathrm{WG}}=\frac{2\pi }{\lambda }n_{eff}H\;$$

where *n_eff_* represents the effective index of the fundamental mode (HE_11_) and H is the propagation length (nanofin’s height). By varying nanofin’s diameter, the effective index of the propagated mode is varied, and thus the 2π phase converge can be obtained with a suitable height of nanofins [36,37]. It should be noted that for this kind of phase modulation, the isotropic structures are always utilized, with square or cylindrical geometry mapping the required phase profile. In contrast, another frequently used phase control method is the geometric phase (also denoted as “Pancharatnam-Berry” phase), where the additional phase is generated by the specific spatial orientation (e.g., rotation angle *θ*) of the anisotropic rotary nanofin to tailor the wavefront of circular polarizations. More specifically, when a circularly polarized light is incident on the dielectric nanofins rotated by an angle of *θ*, the complex transmission coefficient can be expressed by the Jones matrix [38,39]:

(7) $$T=R\left(-\theta \right)*J*R\left(\theta \right)=\left[\begin{array}{cc} \cos\theta & -\sin\theta \\ \sin\theta & \cos\theta \end{array}\right]\left[\begin{array}{cc} t_{xx}e^{{i\varphi }_{xx}} & 0\\ 0 & t_{yy}e^{{i\varphi }_{yy}} \end{array}\right]\left[\begin{array}{cc} \cos\theta & \sin\theta \\ -\sin\theta & \cos\theta \end{array}\right]$$

where *θ* is the rotation angle in the *x*–*y* plane (metalens plane). *R*(*θ*)** and *R*(*−θ*)** are 2 × 2 rotation matrices. *J* is the transmission matrix in the crystal coordinates. *t* and *φ* are the transmission coefficients and structural phase retardation, where the subscripts *xx* and *yy* indicate the polarization direction of the incident beam parallel to the *x* or *y*–direction. With the above equation, the output transmitted field upon a circularly polarized incident beam (E_in_ = [1, *±i*]) can be expressed as:

(8) $$E_{\mathrm{out}}{=T*E}_{\mathrm{in}}=\frac{1}{2}{(t}_{xx}e^{{i\varphi }_{xx}}+t_{yy}e^{i\varphi_{yy}})\left(\frac{1}{\pm i}\right)+\frac{1}{2}{(t}_{xx}e^{{i\varphi }_{xx}}-t_{yy}e^{i\varphi_{yy}})e^{\pm i2\theta }\left(\frac{1}{+i}\right)$$

Clearly from Equation (4), the output electric field consists of two parts, the first item refers to the co-polarized output beam without change of polarization states, and the second item represents cross-polarized (opposite handedness) beam carrying an additional phase Φ = *2θ*, which is known as the PB phase. To achieve 2π coverage by utilizing the geometric phase method, the rotation angle for each nanofin in the metalens plane should satisfy the following equation:

(9) $$\theta (x,y)=\frac{1}{2}\varphi (x,y)$$

where *φ* (*x*, *y*) is the required phase indicated in Equation (5). From Equation (9), it is clear that by continuously rotating the nanofins radially from the center to the edge of the metasurface, a full 2π coverage can be smoothly obtained. It should be noted that the PB phase modulation method only applies to incident lights with circularly polarization (CP) states, and thus there is inevitably polarization conversion which limits the focusing efficiency of the fiber-integrated metasurface. To maximize polarization conversion efficiency, the nanofins should act as half-waveplates by tailoring the dimensions (length, width, etc.) of the nanofins [40,41,42].

**Figure 2:**
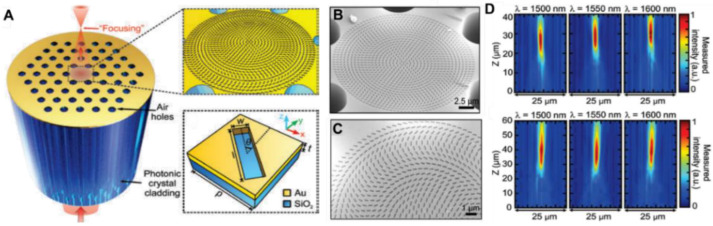
(**a**) Schematic of in-fiber metalens based on LAM-PCF. (**b**,**c**) SEM images of the fabricated PCF metalens with NA = 0.37. (**d**) Measured intensity distributions of PCF metalenses with NA = 0.37 and NA = 0.23. Reprinted with permission from Ref. [43]. Copyright 2019 De Gruyter.

In 2019, Yang et al. first reported the direct combination of optical fiber platform with the plasmonic metasurface for light beam focusing from the fiber output end [43,44]. In this work, the circular gold metalens was directly patterned on the facet of large-mode-area photonic crystal fiber (LAM-PCF) by focused ion beam (FIB) milling. A single etched gold nanorod was considered as the unit element with varying orientation angle (0–164°) radially to construct the hyperbolic phase profile covering 2π indicated in Equation (5), using the geometry phase method with CP incidence. The detailed fiber metalens structure is depicted in Figure 2a–c.

**Figure 3:**
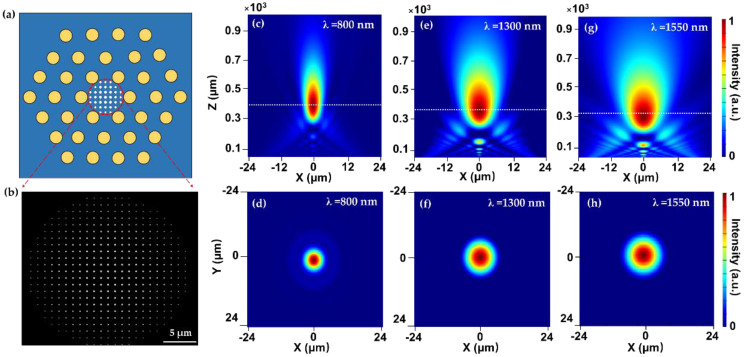
(**a**,**b**) Schematic of all-glass PCF metalens. Normalized intensity distribution of focal spots at x-z and x-y plane upon incident beam at the wavelength of (**c**,**d**) 800 nm, (**e**,**f**) 1300 nm, and (**g**,**h**) 1550 nm. Reprinted with permission from Ref. [46]. Copyright 2021 MDPI.

By both experimental and computational simulation, the proposed LAM-PCF metalens with two different numerical apertures (NA) has demonstrated good focusing performance with focal lengths of 30 and 50 μm upon the incident RCP light at 1550 nm. Furthermore, the enhanced optical intensity has been found to be over 230% due to the tight and bright focusing spot (See Figure 2d). Following Yang’s work, in 2020, Korean researcher Kim et al. suggested that the PCF-based metallic metalens in [43] suffer from a low operation efficiency (~17%) due to the low polarization conversion efficiency and metal loss. As a result, Kim et al. proposed an all-dielectric metalens by depositing the aperiodic silicon (Si) nanopillars on top of the photonic crystal fiber [45]. The focusing effect was realized by tuning the diameters of Si nanopillars using the propagation phase. Simulation results have shown that the focusing efficiency of the dielectric PCF metalens has been improved to 88% with a focal length of 30 μm. Although the operating efficiency of the proposed PCF metalens has been enhanced, however, neither the newly designed fiber platform (the PCF type is the same as reported in [43]) nor the broadband focusing is presented in this work. In this regard, in 2021, Zhao et al. designed a customized all-glass PCF metalens for output guided beam focusing [46]. The schematic is shown in Figure 3a,b.

**Figure 4:**
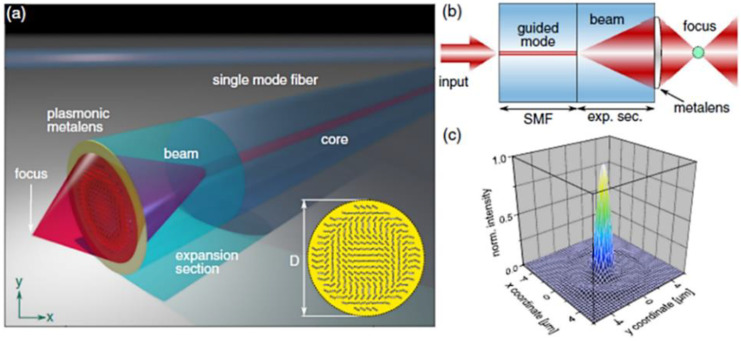
(**a**) Schematic of gold-coated plasmonic metasurface interfacing with single-mode step-index fiber. (**b**) Illustration of the interfacing structure including fiber expansion section. (**c**) Measured transverse intensity distribution of the focused spot in the focal plane. Reprinted with permission from Ref. [49]. Copyright 2021 WILEY-VCH.

Zhao et al. replaced the air-holes constituting PCF cladding with fluorine-doped glass rods to reduce the refractive index between the core and cladding, which increased the single-mode operation regime. The designed LAM-PCF for loading the dielectric metalens has a large core diameter of 50 μm, which is twice as large than that used in [43,45]. The large core size supports more unit cells with higher resolution to tune the phase profile, and most importantly, allows for a larger focal length according to Equation (5). The 2π phase modulation was achieved by varying the diameters of TiO_2_ nanopillars using the propagation phase modulation method. Moreover, the author demonstrated a broadband focusing function with the designed LMA-PCF metalens covering the typical “three communication windows” (800–1550 nm), and the focusing performance with varying incident wavelengths has also been well studied. The simulated results have shown that the customized all-glass LMA-PCF could be operated in the broadband near-infrared range with a stably high focusing efficiency (~70%) and large focal length (~300 μm), which has greatly improved the focusing performance of in-fiber metalens. Besides the PCF as the substrate for integrating the flat metalens, single-mode fibers have also been selected as an appropriate candidate for saddling the metalenses to achieve the short or long-distance focusing of fiber guided mode in either visible band or the near-IR range [47,48,49,50,51,52,53], which is more approachable for the practical applications in the long-haul optical communication systems. Besides the extensive study of the focusing proprieties for this kind of fiber metalens, it has been found that the numerical aperture (NA) is also a key factor affecting the performance of optical since a larger NA supports a higher coupling efficiency of optical fiber to be applied to high-power applications. In this regard, the optical fiber metalens with the purpose of increasing the NA is also studied. Mostly recently, Matthias et al. proposed a model which combines the single-mode fiber with plasmonic metalens via a coreless glass section (expansion section, Figure 4a,b). By means of the insertion of the expansion section, the light propagating through the fiber end could be expanded to 48 μm and thus greatly enlarge the NA of the metalens (~0.3) [49]. In this application, the geometry phase method was applied, with gold nanoslits orientated to different angles to achieve the 2π phase profile indicated in Equation (5). The dimensions of the nanoslits were optimized using the Babinets’ principle to achieve the maximal transmission (T ~ 0.332) at the desired wavelength λ = 650 nm, resulting in L = 140 nm, W = 60 nm. This fiber metalens concept will find applications in a multitude of fields, including remote focusing, optical trapping, beam generation, and efficient light collection.

**Figure 5:**
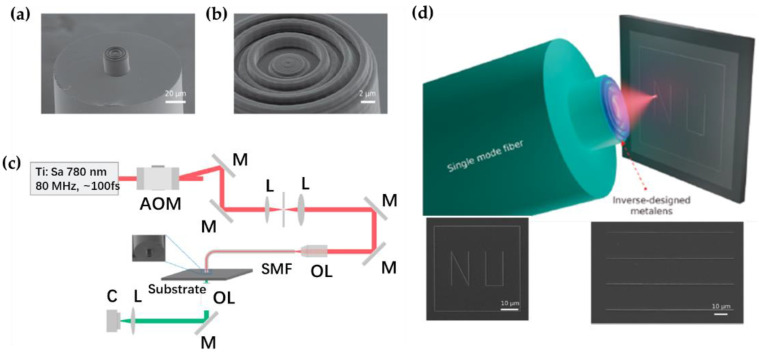
(**a**) SEM image of the fabricated metalens on top of the fiber core. (**b**) Enlarged view of the fiber meta-tip lens. (**c**) Schematic of the homemade two-photon laser writing system. (**d**) Illustration of two-photon writing process via fiber meta-tip lens and with the patterned “NU” and straight lines. Reprinted with permission from Ref. [54]. Copyright 2021 American Chemical Society.

In another practical application with light beam focusing, the fabricated fiber-integrated metalens was self-adapted and was directly applied to the laser lithography system for light beam routing [54]. The circular metalens, made of photoresist, has a phase profile defined by Equation (5), a focal length of 8 μm, and a near-infrared operating wavelength of 980 nm (See Figure 5a,b). 

The inverse-designed was fabricated via 3D direct-writing methodology [55]. The homemade fiber focusing metalens was employed as the objective lens and directly integrated into the two-photon laser writing system for sample patterning (Figure 5c,d). It has been demonstrated that the fiber meta-tip that incorporated two-photon laser writing setup has a better patterning resolution (~200 nm), as compared to the commercial direct laser writing system.

### 3.2. Function of Light Beam Routing 

**Figure 6:**
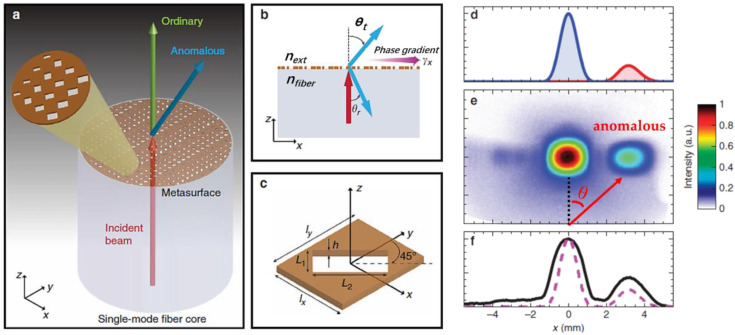
(**a**) Schematic of fiber meta-tip. (**b**) Illustration of generalized Snell’s law described in Equation (6). (**c**) Nanohole as the unit cell with 45° orientation in the x-y plane. (**d**) Simulated electric field-intensity profiles of fiber meta-tip 3 (γ = 14960 rad·cm^−1^). (**e**) Measured field-intensity map at z = 8 mm. (**f**) Transverse cuts at y = 0 comparing the measured (black-solid curve) and simulated (magenta-dashed curve) results. Reprinted with permission from Ref. [57]. Copyright 2017 Nature Publishing Group.

Apart from the light focusing function of optical fiber-integrated metalens, another typical functionality achieved by optical fiber-integrated metasurface is the light beam steering. This function is of great importance because it can not only flexibly control the propagation direction of the output beam, but it can also distinguish the incident beam with different chirality without the use of the bulky optical components (e.g., reflecting mirrors, wave-plates). It is worth mentioning that Maria et al. in 2017 first proposed the single-mode optical fiber meta-tip for beam steering (deflection) with the phase-gradient metasurface [56,57]. The proof-of-concept application was the beam steering of a transmitted beam by an arbitrary deflection angle. The deflection angle of the anomalous refraction, under normal incidence, can be derived from the generalized Snell’s law and Equation (1), which is expressed:

(10) $$\sin\alpha =\frac{\lambda }{{2\pi *n}_{fiber}}\gamma_{x}$$

where α is the deflection angle, *n_fiber_* is the refractive index of the fiber, and γ_x_ is the phase gradient along the x direction in the metasurface plane. By means of Babinet-inverted plasmonic metasurfaces with tuning the rectangle nanoholes’ side lengths, the 2π phase coverage is achieved for the anomalous deflection beam [58]. The phase gradient can be calculated by:

(11) $$\gamma_{x}=\frac{\Delta \emptyset }{l_{x}}$$

where ΔΦ and *l_x_* represent the phase difference and distance between neighbor nanoholes. By varying the side lengths (L_1_ and L_2_) of the nanoholes according to the simulated “look-up” phase map, five prototypes of single-mode fiber-based metalens with varying phase gradients have been fabricated. Upon experimental verification, the anomalous beams with cross-polarization to the incident beam were deflected accordingly from 11° to 22°, as indicated by Equation (10), and the constructed fiber meta-tip prototype and beam deflection performance are depicted in Figure 6a–f.

**Figure 7:**
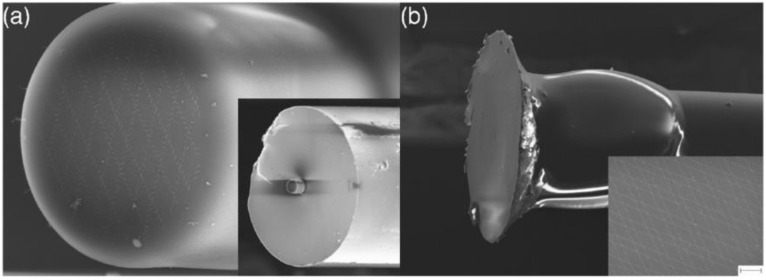
Micrograph images of patterned fibers. (**a**) Image of a polarimeter fabricated with the fiber exposure approach. (**b**) Image of a patterned fiber facet using the flood exposure approach, where a much larger area of nanoantennas arrays is transferred. Scale bar = 2 μm. Reprinted with permission from Ref. [59]. Copyright 2019 IEEE Photonics Society.

Following Maria’s work, in 2018, Michael et al. firstly proposed a UV curable polymer in-fiber polarimeter (see Figure 7a,b) with the template stripping transfer method [59,60]. The reason for using gold nanoholes as the unit cell is that the template stripping method could enhance the adhesion between metals (gold included) and silicon to ensure that the nanostructure pattern is smoothly transferred to the fiber core. The metasurface was made of two superimposed gratings of antenna columns arranged in a pattern where the antennas in each column are rotated 90° relative to antennas in the neighboring column, and the spacing between. The distance between the two columns is set as λ* (1 + 1/4) (λ is the resonant wavelength of the gold antenna) to scattering of polarization-dependent in-plane and out-plane grating orders. The out-plane order was scattered at an angle of 45° from the metasurface plane (λ = 1550 nm) and was used for polarization measurements. The authors have demonstrated the validity of the homemade in-fiber polarimeter since the measurement results of polarization states of the incident beam from the in-fiber polarimeter are almost identical to that of a commercial free-space polarimeter. The integration of in-line polarimeters represents an important step towards the miniaturization of optical polarimeter but is also useful for controlling light polarization in optical communication systems.

**Figure 8:**
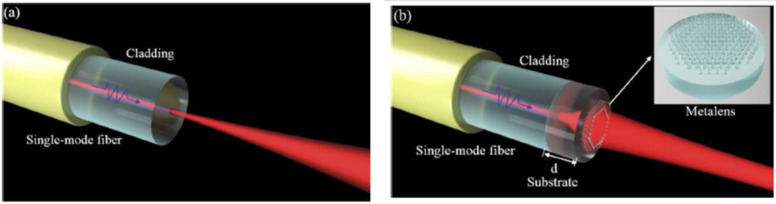
Light propagation through the SMF without (**a**) metasurface on the facet and (**b**) with metalens to control the light beam convergence. Reprinted with permission from Ref. [61]. Copyright 2021 Optical Society of America.

In addition to the beam deflection, the collimation of light beam has also been theoretically studied based on single-mode fiber (SMF600) metalens [61]. The key feature in this research is the use of low refractive index material (polymer, n = 1.52) to form the metalens to reduce the optical impedance match between the metasurface/fiber interface (see Figure 8a,b). More precisely, the elliptical nanopillars with uniform height but varied width and length (100–400 nm) constructed the metalens with 2π phase modulation (as described in Equation (5)), thus collimating the divergent beam output from the fiber end. It has been found that the highly divergent beam from the fiber facet can be tightly collimated with high efficiency of 95% while maintaining the Gaussian beam profile, and the concept of optical fiber metalens collimator may find applications in laser-delivery, biomedicine, and optical imaging.

In view of the abovementioned applications, it is evident that the combination of optical fiber with the superior light-guiding capability from the flat metasurface has provided an unprecedentedly well-established platform for the creation of novel photonic devices with complex functionalities at multiscale, which greatly advances the production of multiple photonic devices to be applied in diverse optical systems.

### 3.3. Function of Biological Sensing and Imaging

The real-time and accurate sensing of multiple physical quantities has never failed to draw attention. Conventional optical fibers have been well explored as a mature platform for multiparametric environmental monitoring. For plasmonic nanosensors based on the electromagnetic resonance, whether they are surface plasmon resonance (SPR) or localized surface plasmon resonance (LSPR), enhancing the light-matter interactions is the most critical way to improve their performance [62]. The enhancement of plasmonic sensing can be achieved with either the optimized design of the nanostructures or the tuning of plasmonic via nanoparticle growth [63]. Meanwhile, the emergence of metasurface technology of controlling the light proprieties with the principle of phase discontinuity has opened up new windows for the sensing and monitoring of environmental variables. Of different fiber sensing platforms, the biomedical applications based on optical fiber metasurface-based devices begin to draw attention from researchers worldwide. 

**Figure 9:**
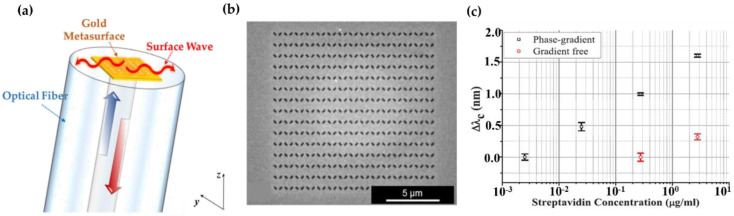
(**a**) Schematic of the fiber meta-tip for the excitation of the surface wave. (**b**) SEM image of the phase-gradient meta-tip. (**c**) Dose-response curves pertaining to the phase-gradient fiber meta-tip (black squares) and gradient-free benchmark (red circles) biosensing platforms. Reprinted with permission from Ref. [65]. Copyright 2020 Wiley-VCH.

Further to the model described in Section 3.2 for the function of beam deflection, Marial Principe et al. has expanded their work into the application of label-free biological sensing [56,64], with the fiber meta-tip named “prototype 5” indicated in [57]. As seen in Equation (10), there exists a critical phase gradient above which the incident plane wave is driven to surface wave (evanescent range, *θ_t_* = 90°, grazing condition), as expressed by:

(12) $${\;\gamma }_{x}\gg n_{t}\frac{2\pi }{\lambda }$$

where *n_t_* is the refractive index of the transmission region. To maximize the phase gradient, the authors use the minimum number of etched gold nanoholes to obtain the maximal phase change ΔΦ = π. Considering the biological experiments (e.g., liquid biologic solution with RI of 1.34) and the operating wavelength (1400–1600 nm), the side lengths (L_1_ and L_2_) have been optimized to locate the resonance wavelength in the operating range as well as fulfilling the grazing condition. Figure 9a,b shows the schematic of fiber meta-tip coupling the anomalous transmitted beam into a surface wave. A Babinet-inverted, plasmonic phase-gradient MS, comprised of rectangular nanoholes milled in a thin gold film, is laid on the flat fiber tip, which is assumed to separate the fiber core (incidence region) and the exterior medium (liquid biological solution). The authors have compared the surface sensitivity of phase-gradient metasurface and gradient-free one by observing the plasmonic resonance wavelength shift of nanoholes under the same local refractive index environment, and the experimental results have shown that the phase-gradient metasurface features a higher surface sensitivity, indicated by a larger wavelength shift and enhanced local field enhancement. The enhanced surface sensitivity of phase gradient fiber metasurface was further demonstrated by the real-time and high-sensitivity monitoring of biological molecules (Streptavidin ~a few ng/mL) [65].

**Figure 10:**
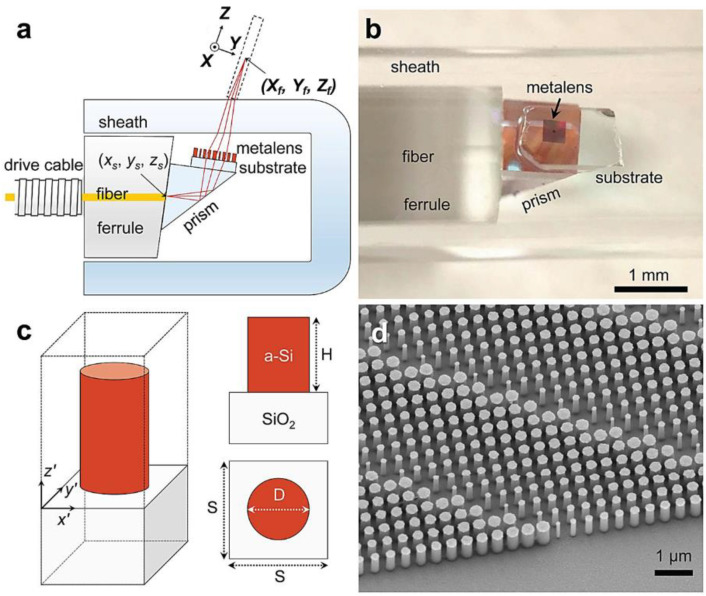
(**a**) Schematic of the nano-optics endoscope. (**b**) Photographic image of the distal end of the nano-optics endoscope. (**c**) Schematic of an individual metalens building block consisting of amorphous silicon (a-Si) nanopillar on a glass substrate. (**d**) SEM image of a portion of a fabricated metalens. Reprinted with permission from Ref. [72]. Copyright 2018 Nature Publishing Group.

By implementation of real-time biological experiments, the phase-gradient fiber meta-tip is capable of detecting the slight concentration change of Biotin and Biotin–Streptavidin interaction, evidenced by a larger resonance wavelength shift (Figure 9c). The wavelength shift is considered as an important parameter in label-free chemical and biological sensing applications. The enhanced sensitivity of fiber meta-tip benefits from the coupling of the incident field to the plasmonic resonance, thus yielding a higher field enhancement. Furthermore, the authors have also figured out the detection limit of the biological molecules (3 nm/mL) with the proposed fiber meta-tip, which is proven to prevail over the fiber optic biosensors with the current state of the art [66,67,68,69,70,71]. Another impressive example of fiber-integrated metalens for biological imaging was reported by Hamid et al. from Harvard Medical School [72]. The metalens (290 × 290 μm^2^), made of silicon pillars, was embedded on the fiber endoscopic catheter (see Figure 10a–d), and could achieve varying near-diffraction-limited tight focal points in response to the incident wavelength (λ = 1.31 μm). This results in effective high-quality axially-shifted imaging for subsurface tissues (lung specimens and sheep airways) in vivo, in which the transverse resolution and the high depth-of-focus have been perfectly balanced.

Obviously, the biomedical applications obtained through the optical fiber-integrated metasurface have opened up intriguing avenues towards the fabrication of the miniaturized plug-and-play optical fiber metasurface-based devices. These fiber meta-devices, with advantages such as excellent light tuning ability, small volume, and even biocompatibility, may find plenty of optical applications in the biomedical and clinical field including real-time biological parameters testing, liquid biopsy, cancer diagnosis, and high-resolution medical in vivo imaging.

### 3.4. Function of Special Beam Generation and Applications for Optical Communication

**Figure 11:**
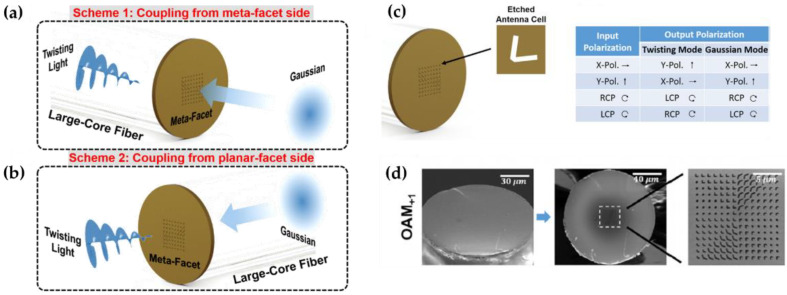
Schematic of the fiber meta-tip for twisting broadband light from either (**a**) meta-facet side or (**b**) planar-facet side. (**c**) Metasurface structure with etched “V-shape” antenna arrays and the conversion relationship between input and output polarization states. (**d**) SEM images of the original fiber facet fabricated on-top metasurface and enlarged metasurface region with OAM_+1_. Reprinted with permission from Ref. [75]. Copyright 2018 AIP Publishing LLC.

Since metasurfaces are capable of controlling light properties (phase, amplitude, polarization, optical impedance, etc.) in 2D versions, they are undoubtedly promising candidates for shaping and generation of nonconventional light beams with special wavefronts (vortex beam, twisted beam) [25,73,74]. When combined with optical fibers, the generation of special light beams could be of great use in a variety of optical applications, including fiber-optic communication, light beam manipulation, and information processing. In 2018, Yifan Zhao et al. first demonstrated a large core optical fiber meta-tip for the generation of twisted light (linearly polarized, circularly polarized) from both the meta-tip side (OAM_+1_) and the planar-facet side (OAM_-1_) [75]. The 2π phase coverage was achieved by judiciously arranging eight etched gold “V” antenna arrays on top of the fiber core (d = 14.6 μm) with continuous change of included angle θ and arm length L (see Figure 11a,b), resulting in a phase shift interval of π/4. The arm width and cell lateral period of each “V” antenna are fixed at 100 nm and 1.4 μm. The plasmonic metasurface was then capable of twisting the incident light with cross-polarized states with a conversion efficiency of ~9%. In addition to the generation of twisting incident light beam, the reconstruction of the phase profile of the generated twisted light has been achieved with the tilted interferogram using the Fourier-transform method, which had a broadband operating wavelength range of 1480 to 1640 nm with a high phase purity beyond 90%.

**Figure 12:**
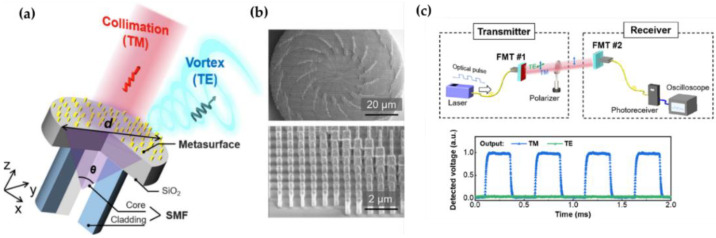
(**a**) Schematic of bifunctional optical fiber meta-tip. (**b**) SEM images of the metasurface and the zoomed-in nanobricks. (**c**) Demonstration of polarization-controlled data transmission with a pair of fiber meta-tips and the transmitted output signals with TE/TM polarization states. Reprinted with permission from Ref. [85]. Copyright 2021 Wiley-VCH.

The results presented in this paper bring new insights for the fiber metasurfaces, since the generated propagation modes can be feasibly applied in fiber-optic communication systems. Indeed, with further consideration of the prototype depicted in [75], it is seen that the created twisted light with OAM ± 1 can further propagate along the fiber for optical data transmission. Furthermore, higher-order OAM modes could also be supported by the replacement of other types of fiber platforms (e.g., few-mode fiber, multimode fiber) with the current metasurface structure. This kind of fiber-integrated meta-device will show great potential in high-speed and mass fiber-optic communication systems, especially those with space-division multiplexing (SDM) technology using OAM modes as the input signal channels [76,77,78,79,80,81,82]. Similar optical fiber meta-tips for the generation of structured light generation and beam shaping can be found in [83,84]. In the meantime, great importance should be attached to the pioneered work demonstrated by Changyi Zhou et al. in 2021, who has successfully built up an all-dielectric metasurface based on a single-mode fiber [85]. This work has overcome the shortcomings of intrinsic ohmic loss encountered by the metallic metasurface structures. Benefiting from the 2π phase coverage from the spatially arranged silicon nanobricks (Figure 12a,b), this all-dielectric fiber meta-tip enables two different functions, with the vortex beam generation from TE-polarized incident beam and the collimation of TM-polarized incident beam. Based on the polarization-selective characteristics of the proposed fiber meta-tip, the authors further reinforce and exploit its practical application in optical systems in which a pair of fiber meta-tips are combined to constitute the optical interconnects for optical data transmission systems (Figure 12c). The optical interconnects acted as an effective channel gate in which the on/off state is strictly dependent on the indent beam polarization state (with the passthrough of TE-polarized mode and blocking of TM-polarized mode).

**Figure 13:**
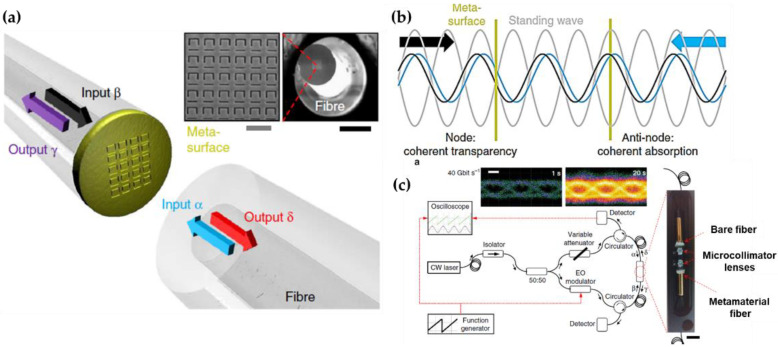
(**a**) Coherent optical input signals α and β interact on a metasurface absorber, generating output signals γ and δ. (**b**) The standing wave formed by the counterpropagating input signal. The metasurface could be placed either the node or anti-node where the optical absorption is suppressed or increased. (**c**) Schematic representation of the fully fiberized experimental setup with a photograph of the packaged fiber meta-device. Scale bar = 5 mm. Reprinted with permission from Ref. [86]. Copyright 2018 Nature Publishing Group.

Coincidentally, the practical use of fiber-integrated metasurface-based in the field of fiber communication networks has been further emphasized in [86]. The metasurface consists of 70-nm-thick gold film perforated with an array of asymmetrically split ring apertures (Figure 13a), which can be placed either the anti-nodes (coherent absorption) or nodes (coherent transparency) to flexibly control the optical absorption of incident light from 0 to 100% (Figure 13b).

The fiber metasurface-based device then could feasibly control the output light intensity depending on the phase difference between the coherent input signals, and thus fulfill the function analog to logic gates (XOR, NOT, AND) by altering the input/output signal phase relations operating at both kHz and GHz bitrates (Figure 13c). The experimental results have manifested the fabricated fiber meta-device as an efficient fiberized switcher to be applied for all-optical signal processing in quantum information networks.

### 3.5. Fabrication Methods of Optical Fiber Meta-Devices

The conventional optical fibers, with their advantages including geometric flexibility, high aspect ratio, and planar cross-sections, have provided a favorable platform for the integration of dielectric or plasmonic metasurfaces. Nevertheless, there remains a big obstacle for the traditional nanofabrication technology to be smoothly applied to the facet of the optical fiber due to the limited size of the optical fiber core. Still, great efforts have been made towards the diversity and maturity of the fiber-integrated meta-devices, from the experimental demonstration to the potential practical applications in the market. Except for the theoretic and numerical studies of optical fiber metasurfaces, the fabrication methods of fiber-based metasurfaces reviewed in this article can be categorized into the following types, which are listed in Table 1: (1) Focus ion beam (FIB) milling: FIB milling is the most frequently applied approach for the planar or 3D nanostructure construction with high precision and resolution. However, the FIB filling technique suffers from limited exposure depth and a time-consuming process that inhibits mass production. Still, it is a preferred fabrication method in which the nanostructures can also be patterned on the sidewalls of optical fiber to construct the metamaterial fibers [87,88,89,90]. (2) Electron-beam lithography (EBL): Similar to FIB milling, the EBL is also a precise fabrication method but with prolonged and complex processing. Different from the direct FIB milling, modifications on the size of the apparatus are usually required since the conventional spin coating and resist processing are performed on big wafers. Several additional measures have been taken to uniformize the resist coating or improve the quality of lithographic patterns for better resolution of imprinted metasurfaces on top of the fiber facet [91,92]. (3) Photolithography: the optical lithography yields higher efficiency and throughputs, especially for the patterning of periodic nanostructures on large scales. However, it inevitably encounters complicated extra procedures, including the resist coating, pattern fixture, and alignment with the optical fiber facet. A more approachable fabrication method is the interference lithography with the ease of complex optical systems. Still, it contains multiple steps and is limited to the periodic arrays of nanostructured patterns where the aperiodic metasurface is not applicable. (4) Nano-transfer technique: Nano-transfer provides an effective way for the transferring of subtle metasurface patterns onto the small fiber tips (e.g., single-mode fibers) where direct nanofabrication is not feasible. The patterning resolution and quality can be soundly maintained during the transfer process. However, the defects may be introduced during the transferring process due to the imperfections of the apparatus, and the transferring process is usually labor-intensive and costly. Nanfang Yu et al. has developed inexpensive nano-transfer techniques with either dry and wet transfer processes named “decal transfer” and “nanoskiving”. These approaches have been manifested to be cost-effective, efficient, and convenient for the transferring of dense, sparse, or interconnected metasurface patterns [93,94,95]. (5) Direct laser writing: by either employing the femtosecond laser pulse or the two-photon polymerization, direct laser writing could also be used for the patterning of nanostructures on top of the fiber facets. Nevertheless, subwavelength structures obtained via femtosecond laser ablation usually encounters relatively low resolution, featuring larger dimensions than the operating wavelength [96,97,98]. Alternatively, the two-photon direct laser writing technique prevails in the aspect of constructing complex three-dimensional (3D) structures which are hardly completed by top-down lithography. The superiorities of two-photon direct laser writing have also enabled complex structure prototyping inside the intra-waveguide structures [99,100].

Vastly different from the abovementioned fabrication methods in which the metasurface patterns are patterned on the drawn optical fiber, the preform-based fiber drawing technique allows for the flexible addition of the metamaterials or microscopic features during the preform assembly and drawing process [101]. As an example, the intra-fiber nanowires have been successfully embedded into the metamaterial preforms with different spatial orientations [102,103]. Although this metamaterial preform is a non-optical fiber device, it is anticipated that this technology could be further adapted for the massive production of optical fiber-based metasurface-based devices with multiple functions at multiscale, controlling optical loss and structural confinement.

## 4. Summary and Future Prospects

The emergence of metasurface technology in the last decade has witnessed the prosperity of optical devices being applied almost everywhere in the optical field. The introduction of phase discontinuities of metasurfaces enables the flexible engineering of the light propagation direction, phase, amplitude, and polarization, and thus a diversity of optical meta-devices has been created to meet the increasing demands in the nanophotonic field. Meanwhile, the rise of the “lab-on-fiber “paradigm has brought disruptive developments for the generation of functionalized optical fibers, which has greatly expanded the application scenarios of conventional optical fibers. In this article, the applications of optical fiber-integrated metasurfaces, which is regarded as an emerging platform from the branch of lab-on-fiber technology, have been reviewed comprehensively. The basic concept and principle of metasurface was been introduced first, which is set as the theoretic basis for the implementation of optical fiber metasurfaces. Depending on the specific phase retardation profile and design methods, the applications with brief physics of optical fiber metasurface-based devices have been categorized and reviewed accordingly, followed by the summary of diverse fabrication techniques for the creation of these fiber-based meta-devices. With the review for the fruitful and novel optical fiber meta-devices, it is believed that the fiber-based metasurfaces show great promising potential in a large number of practical fiber-compatible applications, such as signal processing, long-haul fiber-optic communication, biomedical sensing, endoscopic imaging, optical metrology, and optical storage. Still, there are some challenges and potential research directions for this newly-established photonic platform: (1) One of the salient challenges is the development of a mature fabrication technique with high scalability and cost-effectiveness. Currently, most fiber-based metasurfaces rely on the slow etching process such as FIB milling and EBL, which are not suitable for massive and commercial production. Nanoimprinting and self-assembly may be promising inroads towards future volume production but need further investigation. (2) Nearly all optical fiber meta-devices presented at the current stage are composed of lossy metallic material, which greatly impedes the operating efficiency for the designed functions. Other materials with low loss and high light-matter interactions still need further exploration. (3) The optical fiber metasurfaces at the current stage typically fulfill one specific function with a predetermined phase profile. Bifunctional or multifunctional optical fiber metasurfaces can be further developed by combining the nanostructure arrays with different phase profiles or phase modulation principles on the fiber facet, such as directional beam focusing with polarization detection, simultaneous co-axial or off-axis beam focusing, and light beam collimation with the generation of new structured light. (4) More types of optical fibers can be selected as a promising substrate to be the promising substrates for accommodating the 2D nanostructures, such as multicore fibers, ring-core fibers, multimode fibers, or other microstructured optical fibers, which support the high-order guided modes propagation to be applied to the practical application of fiber-optic communication with SDM technology.

## Figures and Tables

**Table 1:** Summary of fabrication methodologies related to optical fiber meta-devices.

Fabrication Methods	Advantages	Resolution	Scalability	Ref.
FIB milling	Extremely high resolution with low lateral scattering	High < 30 nm	Low	[43,44,56,57,64,65,75,84,86]
EBL	Precise geometry and patterning features	High < 30 nm	Low	[47,49,83,85,104]
Nano-transfer	Prevail at defining nanostructures on small areas	Relatively high	Low	[59,93,94,95]
Photolithography	High throughput; Well controlled features	Low~Medium	Medium	[72,105,106]
Direct laser writing	Superior in shaping 3D structures	Low~Medium	Medium	[53,54]

## Data Availability

Not applicable.
